# Methyllycaconitine Alleviates Amyloid-β Peptides-Induced Cytotoxicity in SH-SY5Y Cells

**DOI:** 10.1371/journal.pone.0111536

**Published:** 2014-10-31

**Authors:** XiaoLei Zheng, ZhaoHong Xie, ZhengYu Zhu, Zhen Liu, Yun Wang, LiFei Wei, Hui Yang, HongNa Yang, YiQing Liu, JianZhong Bi

**Affiliations:** 1 Department of Neural Medicine, Second Hospital of Shandong University, Jinan, China; 2 Institute of Neurology, Shandong University, Jinan, China; 3 Key Laboratory of Translational Medicine on Neurological Degenerative Disease in Universities of Shandong (Shandong University), Jinan, China; The University of Melbourne, Australia

## Abstract

Alzheimer's disease (AD) is a chronic progressive neurodegenerative disorder. As the most common form of dementia, it affects more than 35 million people worldwide and is increasing. Excessive extracellular deposition of amyloid-β peptide (Aβ) is a pathologic feature of AD. Accumulating evidence indicates that macroautophagy is involved in the pathogenesis of AD, but its exact role is still unclear. Although major findings on the molecular mechanisms have been reported, there are still no effective treatments to prevent, halt, or reverse Alzheimer's disease. In this study, we investigated whether Aβ_25–35_ could trigger an autophagy process and inhibit the growth of SH-SY5Y cells. Furthermore, we examined the effect of methyllycaconitine (MLA) on the cytotoxity of Aβ_25–35_. MLA had a protective effect against cytotoxity of Aβ, which may be related to its inhibition of Aβ-induced autophagy and the involvement of the mammalian target of rapamycin pathway. Moreover, MLA had a good safety profile. MLA treatment may be a promising therapeutic tool for AD.

## Introduction

Alzheimer's disease (AD), the most prevalent form of dementia in older adults, is a chronic progressive neurodegenerative disorder [1]. AD patients have severe progressive cognitive dysfunction, memory impairment, behavioral symptoms and loss of independence [2]. According to Alzheimer's Disease Intemational (ADI), at least 35.6 million people had dementia in 2010, with the numbers nearly doubling every 20 years [3]. Many factors contribute to the etiology of AD, elevated amyloid-β peptide (Aβ) and loss of nicotinic acetylcholine receptors (nAChRs) being prominent [4]. Extracellular amyloid plaques, predominantly consisting of Aβ, and intracellular neurofibrillar tangles, formed by hyperphosphorylated tau, are the major pathological hallmarks in the brain of AD patients [5]. Abnormal Aβ protein accumulation represents a key feature and is the triggering mechanism of subsequent cerebral degradation in AD [6].

Aβ is generated predominantly as a 40- or 42-amino acid peptide from amyloid precursor protein (APP) on sequential cleavage by β-secretase and the γ-secretase complex [7]. Aβ_1–42_ has a strong ability to oligomerize to form diffusible dimers and trimers as well as larger oligomers, which induces early synaptotoxic effects and progressive dendritic-spine loss in AD [8]. Aβ_25–35_ is neurotoxic when forming oligomers, which is similar to Aβ_1–42_ [9]. Aβ plays a critical role in the pathogenesis of AD and is associated with energy failure, neuronal apoptosis and neuron loss in the AD brain [10]. The mechanism of Aβ in AD pathogenesis is still unclear. However, suppressing Aβ-induced cytotoxicity has become the focus of much AD research.

Macroautophagy (hereafter referred to as autophagy) is an evolutionarily conserved lysosomal-dependent pathway degrading long-lived or misfolded proteins and damaged organelles. It is an intracellular self-defense process by providing an adaptive strategy for cell survival in eukaryotes [11]. Specific membrane segments elongate, encapsulate part of the cytoplasm, and form double-membrane structures to generate an autophagosome. Autophagosomes become autolysosomes by fusion with endosome or lysosome containing proteases (autophagic maturation), and their inner-membrane and contents undergo clearance [12]. In autophagy studies, LC3 is proposed to act during elongation and expansion of the phagophore membrane. LC3 is cleaved to generate the cytosolic LC3-I with a C-terminal glycine residue, which is conjugated to phosphatidylethanolamine. The lipidated form of LC3 (LC3-II) is attached to both faces of the phagophore membrane but is ultimately removed from the autophagosome outer membrane, which is followed by fusion of the autophagosome with a late endosome/lysosome [13]. Mammalian target of rapamycin (mTOR) is a master controller of autophagy. Activated mTORC1 enhances protein translation by directly phosphorylating 4E-binding protein 1 and p70S6K to negatively regulate autophagy, which is involved in normal physiological processes, including aging, and the pathogenesis of diverse diseases, such as certain types of neuronal degeneration and cancer [14]. Autophagy pathology has been observed in AD. A massive accumulation of autophagic vacuoles was observed in dystrophic neurites in an animal model of AD and in postmortem brains from AD patients, which colocalized intimately with β-secretase complexes, APP, and γ-secretase-derived C-terminal fragment (γ-CTF). Here, autophagy seems to be abnormal because of alteration in the endo-lysosomal pathway, which impairs fusion of autophagosomes with lysosomes [15]. It is indicated that abnormal Aβ-related autophagic vacuoles accumulation may closely cause neuron dysfunction and neuron loss, thereby leading to Alzheimer's neurodegeneration [16], [17].

Neuronal nicotinic acetylcholine receptors (nAChRs) are a family of ligand gated ion channels widely distributed in the human brain. Multiple subtypes of these receptors are involved in a wide range of physiological and behavioral processes, including cognitive enhancement, increased arousal and decreased anxiety and neuroprotection [18]. AD is characterized by a loss of neurons, particularly those expressing nAChRs. The loss of nAChRs has been detected in several regions of the brains of patients with AD, which is thought to underlie memory impairment and cognitive deficits in AD [19]. In our previous study, we found that granulocyte colony-stimulating factor could improve the learning and memory deficits of APP transgenic mice by up-regulating α7nAChR in the brain [20]. Aβ_1–42_ co-immunoprecipitated with α7nAChR in postmortem samples of hippocampal tissue from patients with AD [21]. This suggests that Aβ and α7nAChR may have a high affinity and binding interaction. All the evidences have revealed that α7nAChR may play an important role in the Aβ-induced pathogenic process of AD.

Methyllycaconitine (MLA), a norditerpenoid alkaloid isolated from the seeds of Delphinium brownii, is one of the most potent and specific α7nAChR ligands that bind to neuronal α-bungarotoxin sites. Because of its specific, concentration-dependent, reversible, and voltage-independent antagonism, it could inhibit acetylcholine- and anatoxin-induced whole-cell currents in cultured fetal rat hippocampal neurons [22]. One recent report showed that MLA and the weak (<10%) agonist NS6740 reduced lipopolysaccharide-induced tumor necrosis factor α release, so α7nAChR antagonism may confer anti-inflammatory properties on microglia. As well, antagonism of α7nAChRs may reduce neuroinflammation, which is beneficial to AD [23]. Observations of the crystal structure of a complex between MLA and an AChBP isolated from the salt-water snail, Aplysia californica, revealed that MLA interacted with AChBP at the molecular level [24]. Thus, MLA might affect the pathogenic process of AD caused by Aβ.

In this study, we treated the human neuroblastoma cell line SH-SY5Y cell line with Aβ_25–35_ to observe the neurotoxicity of Aβ_25–35_ and analyzed the role of autophagy in Aβ-induced cytotoxicity. Furthermore, we evaluated the effect of MLA on Aβ-induced cytotoxicity and its underlying mechanisms.

## Materials and Methods

### Reagents, chemicals and the preparation of drugs

Fetal bovine serum (FBS; SH300088.03) and RPMI-1640 (SH30809.01B) were obtained from Hyclone. Amyloid β-protein fragment 25–35 (Aβ_25–35_; A4559), methyllycaconitine (MLA; M168), rapamycin (R0395), dansylcadaverine (MDC; 30432), Hoechst 33258 (B2883), dimethyl sulfoxide (DMSO; D5879) and protease inhibitor cocktail (P8340) were from Sigma-Aldrich. Thiazolyl blue tetrazolium bromide (MTT; 0793) was from Amresco. Aβ_25–35_ was prepared as described [25]. Briefly, Aβ_25–35_ was initially dissolved in double-distilled water to 1 mM. The peptide solution was divided into aliquots and stored at −20°C. Before use, the Aβ_25–35_ solution was incubated at 37°C for 7–10 days to form aggregated diffusible oligomers, then diluted in medium to the indicated concentration. MLA was dissolved in double-distilled water as a stock solution at 1 mM. Then the stock solution was divided into aliquots and stored at −20°C.

### Antibodies

LC3B (2775), p70S6K (9202), p-p70S6K (9206) and Beclin1 (3495P) antibodies were purchased from Cell Signaling Technology. β-actin (sc-47778) antibody was from Santa Cruz Biotechnology. P62 (610833) was from Biosciences.

### Cell culture and drug treatment

The human neuroblastoma cell line SH-SY5Y was purchased from the Cell Resource Center, IBMS, CAMS/PUMC. Cells were cultured in RPMI-1640 supplemented with 10% FBS at 37°C. Cells at 60–70% confluence were treated with concentrations of Aβ_25–35_, MLA, rapamycin or Aβ_25–35_ with or without MLA. Control cells were cultured under normal conditions.

### Cell viability assay

Cells were plated in 96-well plates containing complete medium and cultured for 24 h. Then cells were treated with compounds at the indicated concentrations for specified times. After drug treatment, cell viability was measured by MTT assay [26]. Briefly, 10 µl of the MTT solution (5 mg/mL) was added to each well and incubated for 4 h at 37°C. After removing the supernatant, 100 µL DMSO was added into each well. The absorbance was measured at 570 nm with a microplate reader (Thermo, MULTISKAN MK3, USA). All experiments were repeated 3 times.

### Monodansylcadaverine staining (MDC)

To detect autophagy in SH-SY5Y cells, cells were plated on coverslips in 6-well plates. After 24 h, cells were treated with compounds at the indicated concentrations, fixed with 4% paraformaldehyde for 15 min at room temperature, then stained with MDC (1 µg/mL in phosphate buffered saline [PBS]) at 37°C in the dark, and observed immediately with fluorescence microscopy. To quantify the number of cells with acidic vesicles, cells were seeded into 6-well plates and cultured overnight, then stained with 1 µg/mL MDC at 37°C for 15 min. After incubation, cells were washed with PBS and removed with trypsin-EDTA, resuspended, and analyzed by flow cytometry.

### Apoptosis detection by Hoechst 33258 staining

Hoechst 33258 staining was used to detect apoptotic nuclei. Cells were plated in 24-well plates. After drug treatment, cells were stained with 10 µg/mL Hoechst 33258 for 15 min. After being gently washed with PBS once, cells were observed and photographed under a fluorescence microscopy (NIKON ECLIPSE 90i, LH-M100CB-1, Japan).

### Apoptosis detection by flow cytometry

Cells were plated in six-well plates and incubated for 24 h, exposed to desired concentrations of Aβ_25–35_ for 24 h, then harvested by trypsinization, and washed twice in PBS. After staining with a combination of AnnexinV/fluorescein isothiocyanate (FITC) and propidium iodide (PI) (Annexin V: FITC Apoptosis Detection Kit, BD Pharmingen), cells were immediately analyzed by flow cytometry (FACS Calibur, Becton Dickinson).

### Immunocytochemistry

Immunocytochemical staining was performed as described [27]. Briefly, cells were seeded on cover slips over night. After drug treatment, cells were fixed for 30 min in 4% paraformaldehyde. After blocking, cells were incubated with primary antibody (anti-LC3) overnight at 4°C. After being washed with PBS, cells were incubated with PE-labeled secondary antibodies (1∶500; Invitrogen) at room temperature for 1 h, then counterstained with 4–6-diamidino-2-phenylindole (DAPI) for 10 minutes. Images were obtained by laser scanning microscopy (NIKON ECLIPSE 90i, LH-M100CB-1, Japan).

### Western blot analysis

After treatment, cells were collected and washed gently with PBS twice, then lysed with protein lysis buffer (1% SDS in 25 mM Tris-HCl, pH 7.5, 4 mM EDTA, 100 mM NaCl, 1 mM PMSF, 1% cocktail protease inhibitor). Samples were centrifuged at 12,000 g for 15 min at 4°C, and supernatants were collected. The concentration of the protein was determined by Coomassie brilliant blue protein assay. Equal amounts of protein (50 µg) were resolved by SDS-PAGE and transferred onto nitrocellulose membrane, which was blocked with 5% non-fat dry milk in TBS for 1 h at room temperature, and then incubated with primary antibodies (1∶1000) overnight at 4°C. Membranes were washed and treated with appropriate secondary antibodies for 1 h at room temperature. The immunocomplexes were detected with an enhanced chemiluminescence plus kit.

### Electron microscopy (EM)

Cells were postfixed with 2% osmium tetroxide, followed by an increasing gradient dehydration step with ethanol and propylene oxide. Cells were then embedded in LX-112 medium (Ladd), and sections were cut ultrathin (90 nm), placed on uncoated copper grids, and stained with 0.2% lead citrate and 1% uranyl acetate. Images were examined under a JEOL-1010 electron microscope (JEOL) at 80 kV.

### RNA interference of beclin 1 expression

Cells were seeded in 6-well plates and incubated overnight. Control scramble small interfering RNA (siRNA) or beclin 1-targeted siRNA was transfected by use of Lipofectamine 2000 (Invitrogen) according to the manufacturer's protocol. After 48 h transfection, cells were treated with Aβ_25–35_, then collected and cell lysates underwent immunoblotting for Beclin 1 and LC3 protein level. Cells were also processed for cell viability analysis.

### Data analysis

Data were analyzed by use of SPSS 15.0 (SPSS Inc.). Data were expressed as mean ± SE. Differences between groups were analyzed by t test. P<0.05 was considered statistically significant.

## Results

### Aβ25–35 inhibits the growth of SH-SY5Y cells

**Figure 1:**
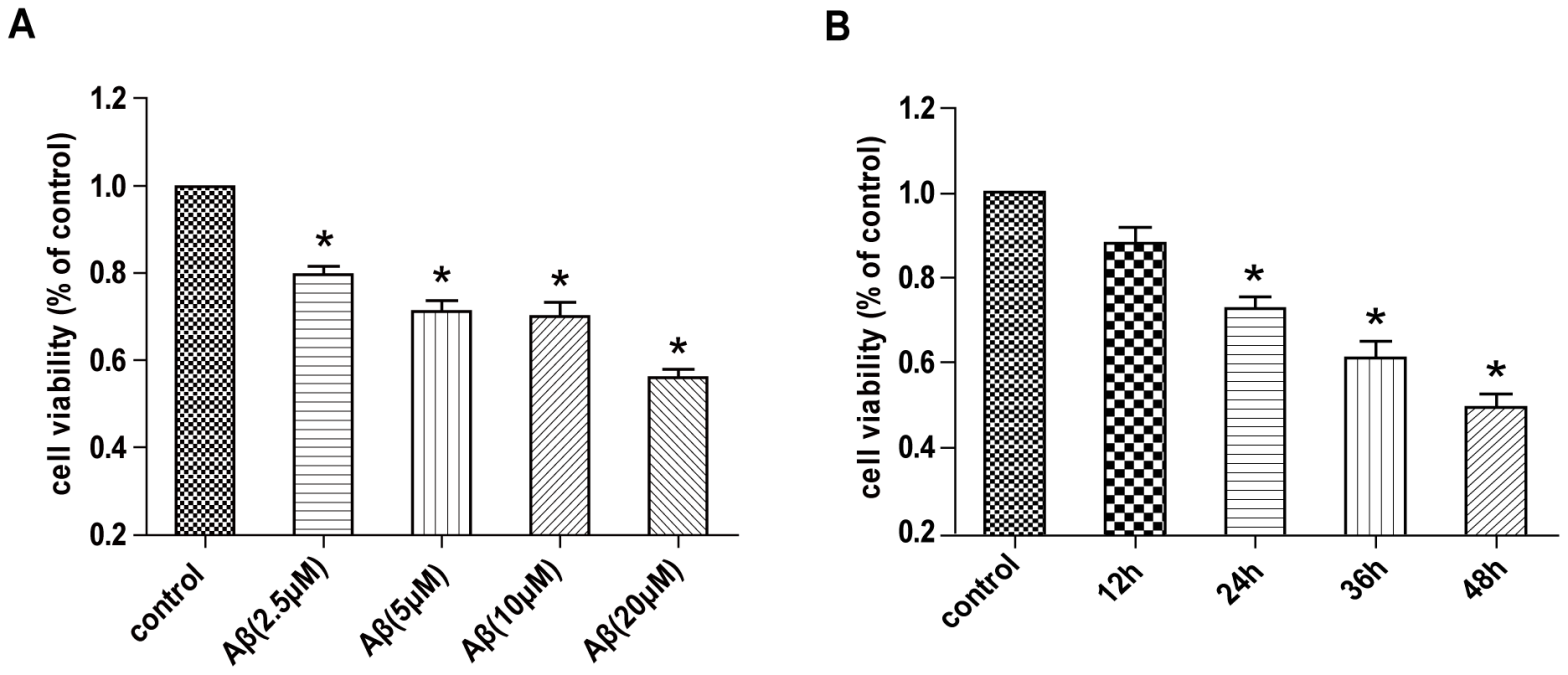
Amyloid-β peptide 25–35 (Aβ_25–35_) inhibits the growth of SH-SY5Y cells. Cell viability was examined by MTT assay in SH-SY5Y cells. Cells were treated with various doses of Aβ_25–35_ for 24 h (A) and 10 µM Aβ_25–35_ for various times (B).

Growing evidence suggests that Aβ has neurotoxic effects both in vitro [28] and in vivo [29], [30], which contributes to cognitive deficits in the pathogenesis of AD. To determine the cytotoxicity of Aβ in SH-SY5Y cells, we pretreated Aβ_25–35_ so that it formed oligomers and then examined the effect of Aβ_25–35_ on the viability of SH-SY5Y cells by MTT assay. Consistent with previous observations [26], we observed the cytotoxic effect of Aβ in SH-SY5Y cells. Cell viability was significantly decreased after exposure to 5, 10 and 20 µM Aβ_25–35_ for 24 h, and cell growth was inhibited by 10 µM Aβ_25–35_ after treatment for 24, 36 and 48 h (Fig. 1A and 1B). Thus, Aβ_25–35_ could inhibit cell growth dose- and time-dependently.

### Aβ25–35 induces autophagy in SH-SY5Y cells

**Figure 2:**
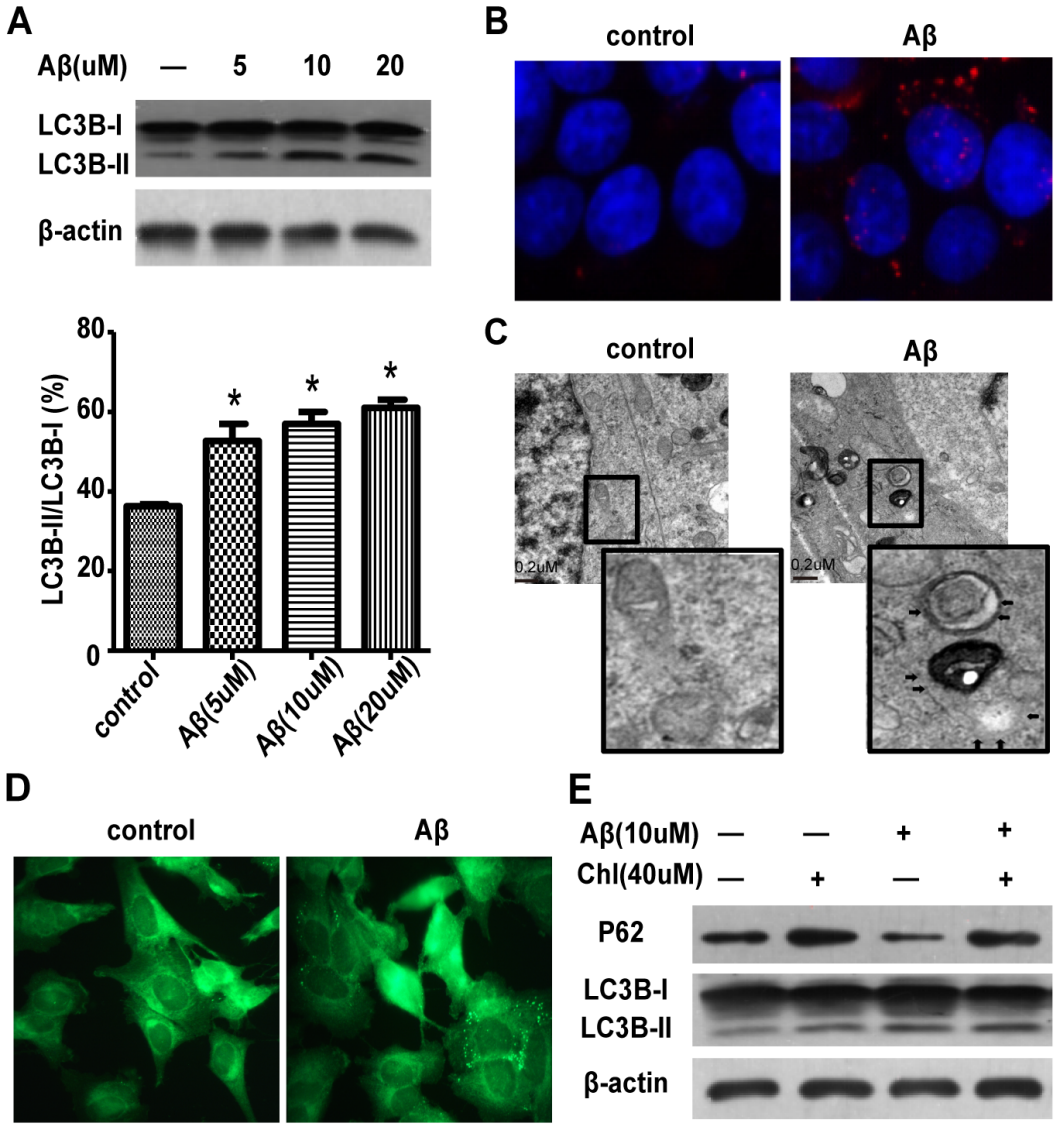
Aβ25–35 induces autophagy in SH-SY5Y cells. (A) Western blot analysis of LC3 protein expression in SH-SY5Y cells treated with different doses of Aβ_25–35_ and quantification, β-actin was a loading control. (B) Immunofluorescence microscopy of punctate pattern of LC3 localization in SH-SY5Y cells treated with Aβ_25–35_. (C) Electron micrographs of SH-SY5Y cells treated with Aβ_25–35_ for 24 h. (D) Fluorescence microscopy of the formation of acidic vesicles after MDC staining in SH-SY5Y cells treated with Aβ_25–35_ for 4 h. (E)Western blot analyses of the protein expression of LC3 and p62 in cells treated with Aβ_25–35_ with and without chloroquine.

Autophagosomes developed in the brains of the AD model mice [31], so we tested whether Aβ_25–35_ could induce autophagy in SH-SY5Y cells. After exposure to exogenous Aβ_25–35_ (5, 10, 20 uM), the level of LC3-II increased in SH-SY5Y cells, and the LC3-II/LC3-I ratio increased (Fig. 2A). To visualize autophagosome formation, the distribution of LC3 was measured by immunocytochemistry with an LC3-specific antibody after administration of Aβ_25–35_ (10 µM). Control SH-SY5Y cells showed a diffuse and weak LC3-associated red fluorescence. The SH-SY5Y cells treated with Aβ_25–35_ exhibited characteristic punctate pattern of LC3, which suggests that Aβ_25–35_ can induce autophagosome accumulation in SH-SY5Y cells (Fig. 2B). Electron micrographs of Aβ_25–35_-treated SH-SY5Y cells also showed abnormal accumulation of large double-membrane vesicles (Fig. 2C), which indicates that Aβ_25–35_ could induce autophagy.

To confirm the cause of Aβ-induced autophagosome accumulation, we analyzed autolysosomal maturation in SH-SY5Y cells with MDC staining. MDC is a lysosomotropic compound used for identifying of acidic vesicles [32]. Thus, MDC staining was used to detect autolysosomes by fluorescence microscopy or flow cytometry. The accumulation of MDC-labeled vacuoles increased with Aβ_25–35_ treatment (Fig. 2D), which suggests that Aβ does not impair autolysosomal maturation. To examine autophagosomal formation in greater detail, we measured the degradation of p62, an autophagy-specific substrate. The degradation of p62 increased in Aβ_25–35_-treated SH-SY5Y cells (Fig. 2E), which suggests that there was no defective clearance of autophagosomes. Thus, Aβ_25–35_ induced autophagy in SH-SY5Y cells, particularly autophagosome formation.

### Aβ25–35-induced growth inhibition of SH-SY5Y cells is mediated by autophagy

**Figure 3:**
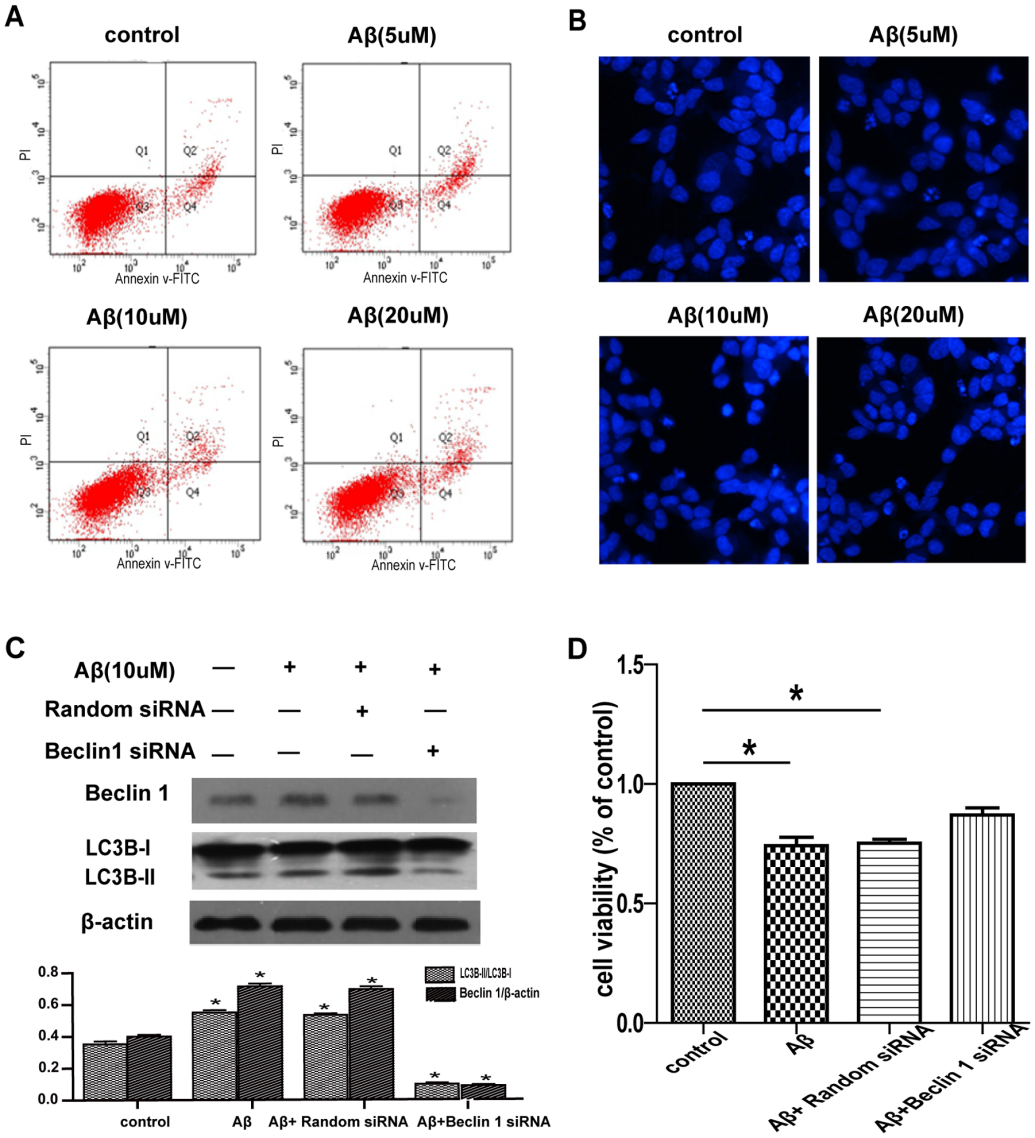
Aβ25–35-induced growth inhibition of SH-SY5Y cells is mediated by autophagy. (A) Annexin V/PI staining of apoptosis of SH-SY5Y cells treated with different doses of Aβ_25–35_ for 24 h. (B) Hoechst staining of apoptosis of SH-SY5Y cells treated with different doses of Aβ_25–35_ for 24 h. (C) Immunoblotting of Beclin 1 and LC3 expression with SH-SY5Y cell lysates. Cells were treated with 10 µM Aβ_25–35_ for additional 24 h after transfection with random siRNA or beclin 1 siRNA for 48 h. (D) MTT assay of cell viability of SH-SY5Y cells treated with 10 µM Aβ_25–35_ for 24 h after transfection with random siRNA or beclin 1 siRNA for 48 h.

To identify the causes of Aβ-induced growth inhibition of SH-SY5Y cells, we tested the effect of Aβ_25–35_ on apoptosis of SH-SY5Y cells by flow cytometry and Hoechst 33258 staining. The SH-SY5Y cells administrated with Aβ_25–35_ for 24 h didn't induce obvious apoptosis (Fig. 3A and 3B). Therefore, cytotoxicity induced by Aβ could not be attributed to apoptosis.

The role of autophagy in Aβ-mediated growth inhibition was further studied by siRNA knockdown of the expression of beclin 1, a component of the class III phosphatidy-linositol 3-kinase complex essential for autophagosome formation. The expression of beclin 1 was markedly suppressed in SH-SY5Y cells transfected with beclin 1 siRNA but not scramble siRNA (Fig. 3C). Accordingly, siRNA knockdown of beclin 1 expression reduced LC3-II accumulation after Aβ treatment as compared with the siRNA control (Fig. 3C). Although the result did not reach statistical significance, it showed that the growth inhibition effect of Aβ could be decreased by siRNA knockdown of beclin 1 expression as compared with the siRNA control (Fig. 3D, P = 0.0522). These data suggest that autophagy is required for Aβ-induced growth inhibition.

### MLA alleviates the toxic effect of Aβ25–35 in SH-SY5Y cells

**Figure 4:**
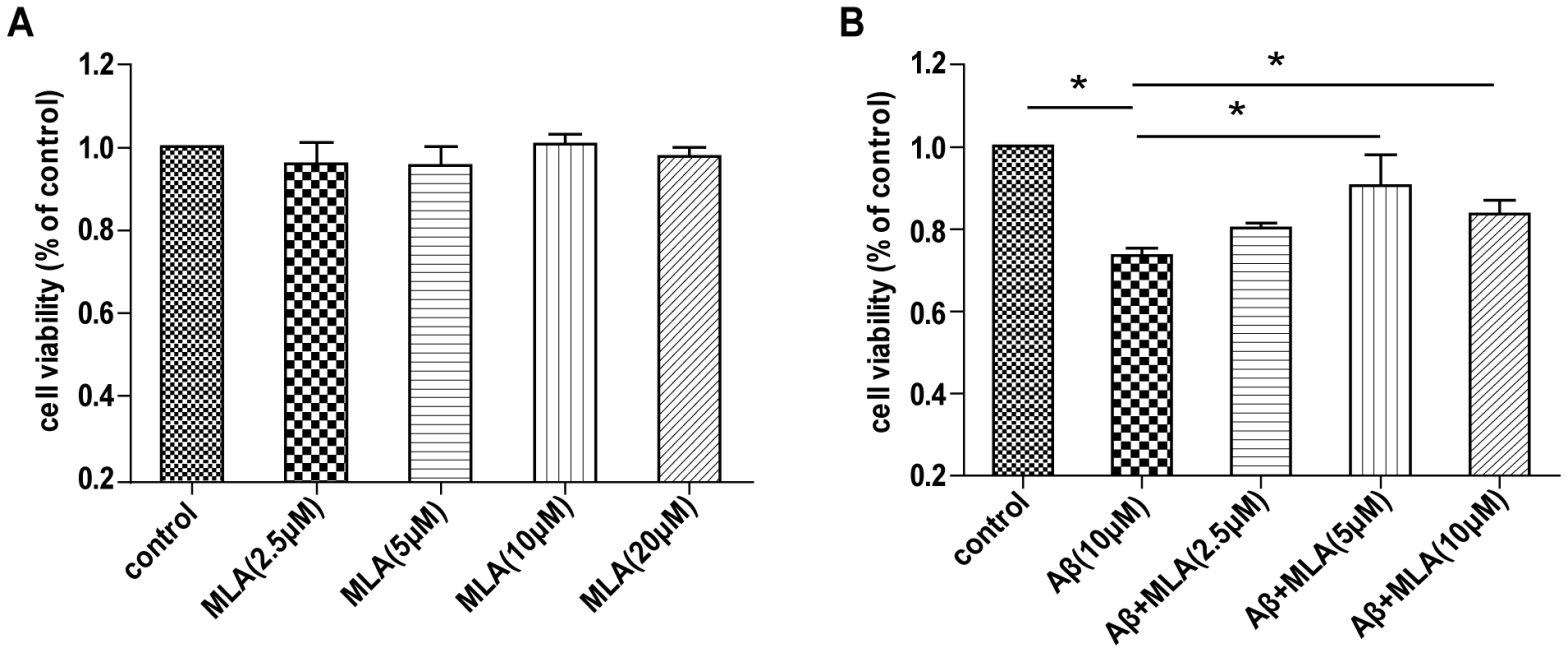
MLA alleviates the toxic effect of Aβ25–35 in SH-SY5Y cells. MTT assay of cell viability of SH-SY5Y cells treated with (A) various doses of MLA for 24 h or (B) 10 µM Aβ_25–35_ with various doses of MLA.

Many studies have shown that α7nAChR could be an important therapeutic target for treatment of AD, because α7nAChR is involved in Aβ-induced neurotoxic effects both in vitro and in vivo [18], [19]. Here, we studied the effect of MLA, a selective α7nAChR antagonist, on Aβ-induced neurotoxicity in SH-SY5Y cells. Pretreatment with 5 and 10 µM MLA inhibited the decreased cell viability induced by Aβ_25–35_ (Fig. 4B), which suggested that MLA had a protective effect against Aβ-induced cytotoxicity. Furthermore, cell viability did not decrease after exposure to MLA (2.5, 5, 10, 20 uM), which suggests a good safety profile (Fig. 4A).

### MLA inhibits Aβ-induced autophagy in SH-SY5Y cells

**Figure 5:**
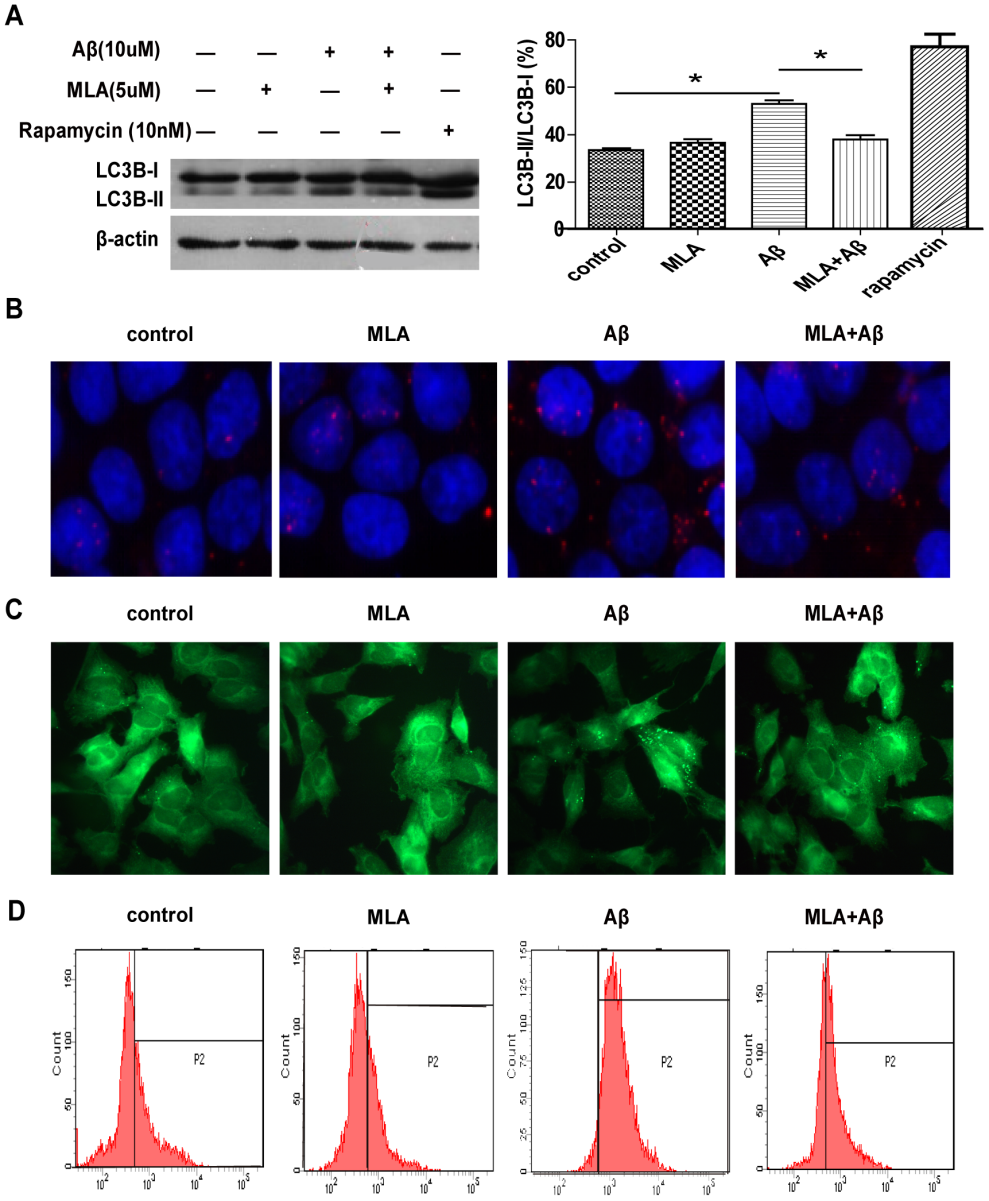
MLA inhibits Aβ-induced autophagy process in SH-SY5Y cells. (A) Western blot analysis of the protein level of LC3 in SH-SY5Y cells treated with 10 µM Aβ_25–35_ with or without MLA. (B) Immunofluorescence microscopy of punctate pattern of LC3 localization in SH-SY5Y cells treated with 10 µM Aβ_25–35_ with or without MLA. (C) Fluorescence microscopy of the formation of acidic vesicles with MDC staining in SH-SY5Y cells treated with 10 µM Aβ_25–35_ with or without MLA. (D) Flow cytometry of the formation of acidic vesicles after MDC staining in SH-SY5Y cells treated with 10 µM Aβ_25–35_ with or without MLA.

To determine the mechanism by which MLA treatment improves the viability of SH-SY5Y cells exposed to Aβ_25–35_, we examined the effect of MLA on Aβ-induced autophagy. Aβ_25–35_ treatment increased LC3-II levels, which was inhibited by administration of MLA (Fig. 5A). To visualize autophagosome formation, LC3 protein was measured by immunocytochemistry with an LC3-specific antibody (Fig. 5B). MLA also inhibited Aβ-induced autophagosome accumulation in SH-SY5Y cells. Furthermore, MDC staining was used to evaluate the effect of MLA on the accumulation of acidic vacuoles induced by Aβ_25–35_. The result showed that Aβ_25–35_ treatment increased MDC-labeled vacuoles, which was inhibited by administration of MLA (Fig. 5C). Flow cytometry also demonstrated decreased MDC-labeled vacuoles with MLA treatment (Fig. 5D). Therefore, MLA could inhibit Aβ-induced autophagy in SH-SY5Y cells, which may contribute to alleviating the cytotoxic effect of Aβ_25–35_.

### MLA inhibition of Aβ-induced autophagy is mediated by mTOR signaling in SH-SY5Y cells

**Figure 6:**
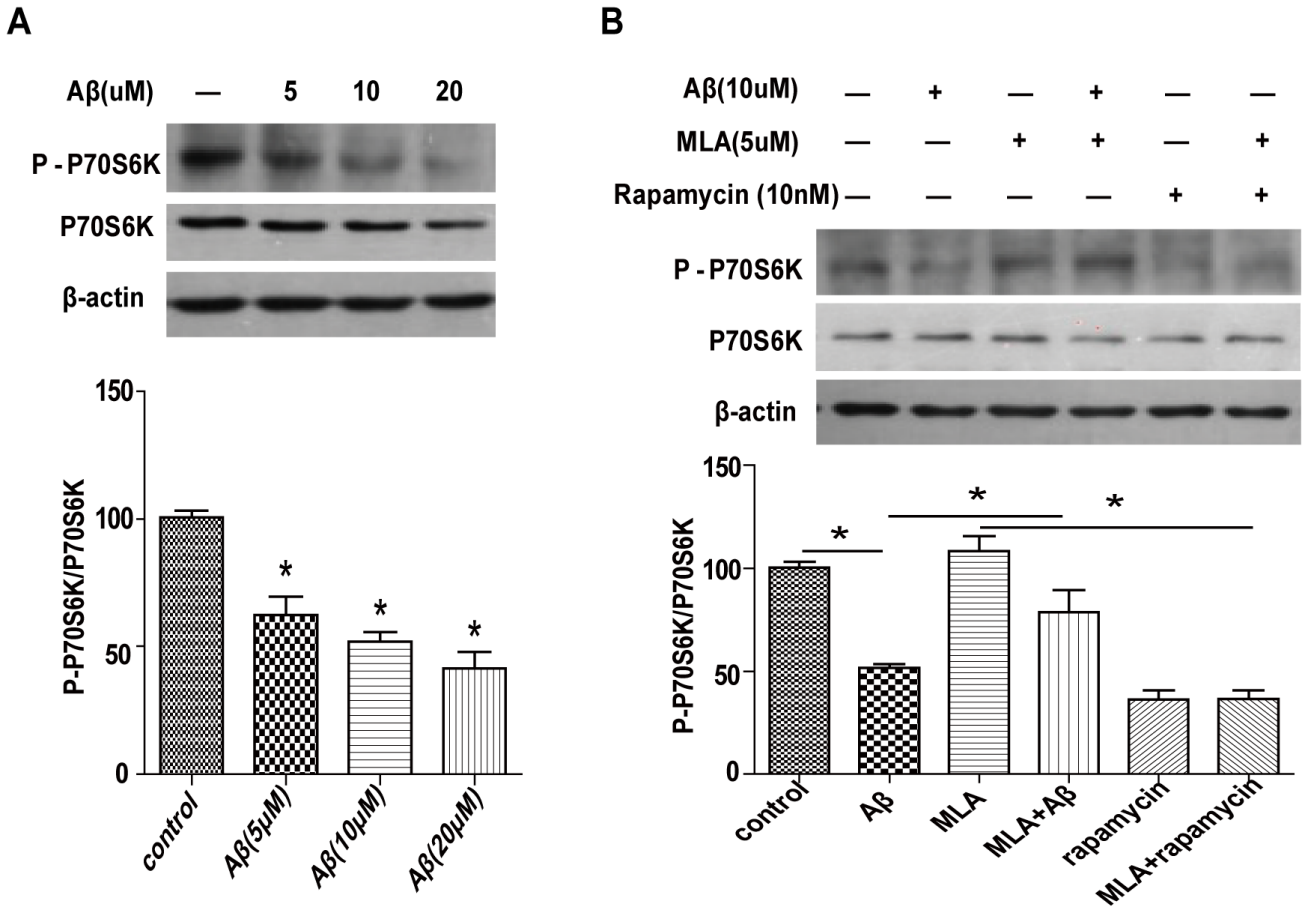
MLA inhibition of Aβ-induced autophagy is mediated by mTOR signal pathway in SH-SY5Y cells. (A) After exposure to different doses of Aβ_25–35_, the level of phosphorylated p70S6K, a mTOR complex 1 substrate, was evaluated by western blot analysis. Signals were quantified by densitometry. (B) Western blot analysis of level of phosphorylated p70S6K after exposure to 10 µM Aβ_25–35_ with or without MLA.

In recent years, several signaling pathways were found to regulate autophagy, with the mTOR pathway playing a key role [11]. We measured the phosphorylation of P70S6K, an mTORC1 downstream substrate, to identify the signaling pathways mediating Aβ-induced autophagy. Aβ_25–35_ treatment dose-dependently decreased the phosphorylation of p70S6K (Fig. 6A). However, Aβ-decreased p70S6K phosphorylation was attenuated by administration of MLA (Fig. 6B), which may contribute to suppress Aβ-activated autophagy. Therefore, MLA may inhibit Aβ-induced autophagy via an mTOR signaling pathway in SH-SY5Y cells.

## Discussion

One of the main histopathological features of AD is the presence of extracellular proteinaceous deposits in the brain, identified as senile plaques, which are enriched in Aβ. It is widely accepted that AD onset can be initially triggered by interaction of Aβ oligomers with the brain parenchyma [33]. In agreement with this, the levels of soluble Aβ oligomers appear to correlate well with the severity of AD dysfunction [34], [35]. In vitro [28] and in vivo [29], [30] studies have shown that these soluble oligomers produced toxicity leading to neuron dysfunction or loss. Consistent with previous studies, we also observed the neurotoxicity of Aβ oligomers on the SH-SY5Y cells. Cell growth was remarkably inhibited by Aβ oligomers treatment. MLA, a norditerpenoid alkaloid isolated from the seeds of Delphinium brownii, had a protective effect against the cytotoxity of Aβ, which may be related to its inhibition of Aβ-induced autophagy and the involvement of the mTOR pathway. Moreover, it had a good safety profile. MLA treatment may be a promising therapeutic tool for AD.

Although AD has been discovered for 100 years, the disease continues to affect millions of patients. Although multiple drugs have now been approved, their expected benefits are modest. Therefore, numerous efforts have been made to develop more potent AD drugs. To date, emphasis has been on strategies to reduce the pathogenicity of amyloid-β (Aβ) peptides [36], widely believed to play a key role in AD. As an antagonist selective for α-bungarotoxin-sensitive α7nAChR, MLA alleviated Aβ-induced cytotoxicity in our SH-SY5Y cells. Pretreatment with MLA could significantly inhibit the decreased cell viability induced by Aβ_25–35_, which indicates a protective effect of MLA against Aβ-induced cytotoxicity. Of note, MLA from 5 to 20 µM alone did not have any significant anti-proliferative effect on SH-SY5Y cells. MLA is a relatively small reversible-binding compound that can easily across the blood-brain barrier in vivo [37], [38]. Considering the low cytotoxicity and the ability to pass the blood-brain barrier, MLA may be a potent drug in the treatment of AD.

Autophagy is a lysosome degradation process that turns over cytoplasmic materials and helps the cell maintain homeostasis. It is usually maintained at low levels under normal conditions for cell survival but can be augmented rapidly as a cytoprotective response when cells undergo starvation or damaging components, such as oxidative stress, infection, or protein aggregate accumulation [11], [12]. Dysregulated or excessive autophagy can lead to cell death. Autophagosomes accumulate abnormally in the brain in several neurodegenerative disorders including AD [39]. Here, we found that Aβ could induce autophagy in SH-SY5Y cells. Aβ treatment could increase LC3II expression, punctate fluorescent signals in SH-SY5Y cells, the formation of acidic vesicular organelles and the accumulation of autophagosomes. These results are consistent with our previous study of PC12 cells in vitro [40].

To investigate the possible mechanism by which MLA pretreatment alleviated the cytotoxity of Aβ in SH-SY5Y cells, we examined the effect of MLA on Aβ-induced autophagy. Aβ-induced upregulation of LC3BII levels and accumulation of autophagosomes or autolysosomes was inhibited by administration of MLA, which suggests that MLA could inhibit Aβ-induced autophagy in SH-SY5Y cells. Autophagy can be a pro-survival response or contribute to cell death; whether it is detrimental or protective remains unclear in AD [41]. It was reported that beclin-1, a protein with a key role in autophagy, was decreased in level in patients with AD and in APP transgenic mice early in the disease process, beclin 1 deficiency disrupted neuronal autophagy and promoted neurodegeneration in mice [42]. Another report showed that rapamycin administration could reduce Aβ levels in neurons and improve cognitive deficits by enhancing autophagy [43]. This evidence indicates that increasing autophagy may be helpful to AD. However, excessive Aβ can activate autophagy, thus resulting in cell dysfunction or death in vitro [44] and in vivo [45], [46], and suppression of autophagy may alleviate Aβ-induced cell death or cognitive deficits. Aβ generation was found linked to autophagy, which is activated and abnormal in AD, and suppressing autophagy by 3-MA could decrease Aβ_1–40_ secretion [47]. Furthermore, an AD drug, galanthamine hydrochloride, and an AD drug candidate, Ghrelin, could inhibit autophagy, which suggests that decreasing input into the lysosomal system may help reduce cellular stress in AD [48]. This evidence suggests that augmented autophagy in AD may be harmful; suppressing augmented autophagy could be an effective therapy for AD. Here, we found that MLA could inhibit Aβ-induced autophagy in SH-SY5Y cells. The suppression of autophagy by MLA may contribute to its protective effect against the cytotoxity of Aβ.

Several signaling pathways regulate the autophagy process with the mTOR pathway playing a key role. We paid attention to the downstream targets. 4E-binding protein 1 and p70S6K are directly phosphorylated by activated mTORC1 to negatively regulate autophagy [14]. In our previous study, we showed that Aβ could induce autophagy in PC12 cells through an mTOR-dependent pathway [40]. Here, we also found that Aβ induced autophagy in SH-SY5Y cells via mTOR signaling as evidenced by the downregulation of phosphorylated p70S6K levels. Moreover, Aβ-decreased p70S6K phosphorylation was attenuated by administration of MLA. The upregulation of mTOR signaling by MLA may inhibit Aβ-induced autophagy and contribute to its protective effect against Aβ-related cytotoxicity.

In conclusion, we showed that Aβ_25–35_ inhibited SH-SY5Y cell growth and induced autophagy. Furthermore, MLA could provide neuroprotection against the cytotoxity of Aβ, which may be related to its inhibition of Aβ-induced autophagy via an mTOR pathway. MLA may be a safe and promising drug candidate for treatment of AD.
